# Chronic Conditions and Food Insecurity in US Children

**DOI:** 10.1001/jamanetworkopen.2025.33953

**Published:** 2025-09-26

**Authors:** Nina E. Hill, Deepak Palakshappa, Kao-Ping Chua

**Affiliations:** 1 Susan B. Meister Child Health Evaluation and Research Center, Department of Pediatrics, University of Michigan Medical School, Ann Arbor; 2 Department of Internal Medicine, University of Michigan Medical School, Ann Arbor; 3 National Clinician Scholars Program at the Institute for Healthcare Policy Innovation, Ann Arbor, Michigan; 4 Department of Internal Medicine, Wake Forest University School of Medicine, Winston-Salem, North Carolina; 5 Department of Pediatrics, Wake Forest University School of Medicine, Winston-Salem, North Carolina; 6 Department of Epidemiology and Prevention, Division of Public Health Sciences, Wake Forest University School of Medicine, Winston-Salem, North Carolina; 7 Department of Health Management and Policy, University of Michigan School of Public Health, Ann Arbor

## Abstract

**Importance:**

Food insecurity is associated with adverse health outcomes in children. There are few recent national studies comparing the prevalence of food insecurity among children with and without chronic conditions.

**Objective:**

To compare the prevalence of food insecurity in children with and without chronic conditions.

**Design, Setting, and Participants:**

This cross-sectional study included children ages 2 to 17 years in the 2019-2023 National Health Interview Survey (NHIS). Data were analyzed December 2024 through February 2025.

**Exposure:**

Reporting any of 7 chronic conditions included in the NHIS: asthma, attention-deficit**/**hyperactivity disorder, autism spectrum disorder, developmental delay, intellectual disability, learning disability, and prediabetes or diabetes.

**Main Outcomes and Measures:**

Food insecurity, defined as low or very low food security in the preceding 30 days. We calculated the prevalence of food insecurity among children with and without any of the 7 chronic conditions, both across the study period and by year. To evaluate whether any differences reflected confounding by demographic characteristics, logistic regression models were fitted that controlled for family characteristics (income, education, number of children and parents, number of employed adults, urbanicity, census region) and child characteristics (age, sex, self-reported race and ethnicity, disability, health insurance type).

**Results:**

Analyses included a weighted sample of 63 163 342 children, of whom 51.1% (95% CI, 50.5%-51.7%) were female with a mean (SD) age of 9.6 (4.6) years. The most common conditions were ADHD (8.6%; 95% CI, 8.2%-9.0%) and asthma (7.0%; 95% CI, 6.7%-7.3%). Across the study period, the weighted prevalence of food insecurity among children with and without chronic conditions was 14.8% (95% CI, 13.7%**-**16.0%) and 9.0% (95% CI, 8.5%**-**9.5%), respectively. This difference was attenuated but persisted after controlling for family and child characteristics (average marginal effect, 2.6 percentage points; 95% CI, 1.7 to 3.5 percentage points). In 2021, the prevalence of food insecurity among children with and without chronic conditions decreased to 9.7% (95% CI, 8.1%-11.7%) and 6.6% (95% CI, 5.8%-7.6%), respectively, but then increased to 15.9% (95% CI, 13.9%-18.2%) and 11.1% (95% CI, 10.1%-12.1%), respectively, by 2023.

**Conclusions and Relevance:**

In this cross-sectional analysis, children were more likely to have food insecurity if they had chronic conditions than if they did not. This difference was only partially explained by several key demographic characteristics. Findings suggest children with chronic conditions should be prioritized in efforts to screen for food insecurity and efforts to enroll patients in programs designed to mitigate food insecurity.

## Key Points

**Question:**

Is food insecurity more common in children with chronic conditions?

**Findings:**

In this cross-sectional study of 2019-2023 data from the National Health Interview Survey, the prevalence of food insecurity was higher among children ages 2 to 17 years with any of 7 chronic conditions compared with those without any of these conditions. This difference persisted even when controlling for several key factors that are associated with chronic conditions and food insecurity, such as income.

**Meaning:**

These results suggest that children with chronic conditions should be prioritized in efforts to screen for food insecurity and efforts to enroll patients in programs designed to mitigate food insecurity.

## Teaser

This cross-sectional study uses 2019 to 2023 National Health Interview Survey data to examine the prevalence of food insecurity among US children with chronic conditions.

## Introduction

Food insecurity is common in households with children, affecting 17.9% of US households with children in 2023.^1^ Food insecurity is associated with several adverse child health outcomes, including asthma, anemia, and poor mental health.^2,3,4,5^ National nutrition support programs, such as the Supplemental Nutrition Assistance Program (SNAP) and special Supplemental Nutrition Program for Women, Infants, and Children (WIC) reduce food insecurity and mitigate its negative effects.^6,7^ In addition, health systems and health insurers are increasingly implementing interventions to assist food insecure patients. These health care-based interventions are termed “Food is Medicine,” and directly provide nutritious foods in the form of produce prescriptions, medically tailored groceries, and medically tailored meals.^8,9^

To optimize the allocation of limited resources for Food is Medicine interventions and other interventions to mitigate the harms of food insecurity, it is important to understand which children are at highest risk for food insecurity. Prior research has linked childhood food insecurity to specific demographic characteristics, such as household income, family size, educational and employment status of adults in households, and receipt of supplemental income for disability.^1^ However, the association between childhood chronic conditions and food insecurity is less clear owing to limitations in existing research. First, many prior studies have examined the association between individual chronic conditions and food insecurity, but few have examined this link across a variety of chronic conditions in children.^10^ Additionally, no prior studies have examined the relationship between childhood chronic conditions and food insecurity over time.

Using nationally representative data from 2019 to 2023, we compared the prevalence of food insecurity in US children with and without 7 common chronic conditions, both across the study period and by year. Additionally, we examined whether any differences in prevalence persisted after controlling for several key potential confounders.

## Methods

### Data Sources

From December 15, 2024, through February 28, 2025, we conducted a cross-sectional analysis of the 2019-2023 National Health Interview Survey (NHIS).^11^ The NHIS is a prospectively collected, nationally representative survey of US households. Households were selected through a stratified, randomized, multistage, probability-cluster design. In each household with children ages 0 to 17 years, 1 adult and 1 child are sampled. Sample weights are then applied, which allow the generation of nationally representative estimates of the US population of noninstitutionalized children. Annualized averages of sample weights are used for the pooled analysis, where individual yearly sample weights are used for trends analysis. Because NHIS underwent a redesign after 2018, the study period began in 2019. Additional details about the NHIS survey methodology have been published elsewhere.^12^

Because data are deidentified, this study was exempted from human participants review by the institutional review board of the University of Michigan Medical School, with informed consent not required. This manuscript follows the Strengthening the Reporting of Observational Studies in Epidemiology (STROBE) reporting guideline for reporting cross-sectional studies.

### Sample

We included children ages 2 to 17 years sampled for the NHIS. The 2019-2023 NHIS includes questions asking whether children in this age range currently have any of 7 chronic conditions: asthma, attention-deficit**/**hyperactivity disorder, autism spectrum disorder, developmental delay, intellectual disability, learning disability, and prediabetes or diabetes (eTable 1 in Supplement 1). These 7 conditions were the only chronic conditions surveyed annually in children during the study period. Because only select conditions were assessed in children age 1 year and younger, we excluded children under 2 years of age. Children were excluded if they had missing data for any of these 7 conditions or any covariate. This complete case analysis approach was unlikely to result in bias because the amount of missing data was small (eTable 3 in Supplement 1).

### Study Variables

The exposure was an indicator that equaled 1 if children had any of the 7 conditions and 0 if they had none of these conditions. The outcome was an indicator for food insecurity. The NHIS includes the USDA 10-item Food Security Survey Module, which assesses household food security in the preceding 30 days using 10 yes or no questions. Questions are completed by the adult household respondent. The NHIS includes a categorical variable based on the number of affirmative responses (0, high food security; 1-2, marginal food security; 3-5, low food security; 6-10, very low food security). We dichotomized this variable into low or very low food insecurity vs high or marginal food insecurity.

### Statistical Analyses

We used descriptive statistics to calculate the prevalence of food insecurity among children with any vs none of the 7 conditions, both overall and by survey year. To evaluate whether any differences reflected confounding by demographic characteristics, we fitted a multivariable logistic regression model in which we modeled the occurrence of food insecurity as a function of having any of the 7 conditions, controlling for family characteristics (income relative to federal poverty level, highest education attainment, number of children, number of parents, number of employed adults, urbanicity, census region), and child characteristics (age, sex, self-reported race and ethnicity, receipt of disability benefits, health insurance type). Census regions included Northeast, Midwest, South, and West. Educational level was defined as the highest educational attainment of the adults in the sample child’s family. Health insurance type was defined as private only, public only, other coverage, or uninsurance. Race and ethnicity were self-reported as non-Hispanic American Indian or Alaska Native, non-Hispanic Asian only, non-Hispanic Black or African American only, Hispanic, non-Hispanic White only, or other single or multiple races. Race and ethnicity were included because prior studies have demonstrated racial and ethnic disparities in both childhood chronic conditions and food insecurity.^13,14^ Disability was measured based on the receipt of Supplemental Security Income or Social Security Disability Insurance. To facilitate interpretation of coefficients as absolute percentage point differences in probability, we calculated the average marginal effect (AME) of having a chronic condition on the risk of food insecurity.^15^ We did not include interaction terms between the indicator for having any of the 7 conditions and covariates, as it was beyond the scope of this study to evaluate whether the association between food insecurity and having these conditions varied by family and child characteristics. The threshold for statistical significance was α < .05 in 2-sided tests.

#### Subgroup Analyses

We conducted 2 sets of subgroup analyses. First, we compared the unadjusted and adjusted prevalence of food insecurity among children with each of the 7 chronic conditions to children without any of these conditions. Second, we compared the unadjusted and adjusted prevalence of food insecurity among children with 1, 2, or 3 or more chronic conditions vs those without any chronic condition. In both sets of subgroup analyses, covariates were the same as the main analysis.

Data processing was performed in SAS version 9.4 (SAS Institute), and statistical analyses were performed in Stata 18/SE (StataCorp). All analyses accounted for the complex survey design of the NHIS using weights and design-based variance estimators.

#### Supplemental Analyses

In one supplemental analysis, we evaluated whether results changed when controlling for the presence of any chronic condition among the adult sampled in the household. This adult was the sampled child’s parent or guardian in 93% of cases. This supplemental analysis excluded children who could not be linked to a sampled adult via the household identifier.

In a second supplemental analysis, we included children with anxiety and depression symptoms as chronic conditions, thus increasing the total number of chronic conditions to 9. These conditions were measured based on having daily vs less than daily symptoms of anxiety and depression. We compared the unadjusted and adjusted prevalence of food insecurity among children with any of the 9 chronic conditions vs those with none of the 9 conditions. We limited this analysis to children ages 5 to 17 years because NHIS does not assess anxiety and depression symptoms for children younger than 5 years.

## Results

### Sample Characteristics

There were 34 188 children ages 2 to 17 years in the 2019-2023 NHIS, corresponding to a weighted annual sample of 65 103 073 children. Of this total, 1001 (2.9%) were excluded due to missing data for chronic conditions or covariates, leaving 33 187 children (weighted annual sample, 63 163 342 children). Of the 63 163 342 children, 12 915 671 (20.4%) had any of the 7 chronic conditions and 50 120 942 (79.4%) had none. Children with chronic conditions were more likely to be female (61.1% [95% CI, 59.7%-62.5%] vs 48.5% [95% CI, 47.8%-49.2%]) and to have Medicaid or other public insurance coverage (45.2% [95% CI, 43.5%-46.8%] vs 34.6% [95% CI, 33.3%-36.0%]) compared with those without a chronic condition (Table 1). Households that included children with chronic conditions were more likely to have income less than 100% of the federal poverty limit (20.0% [95% CI, 18.6%-21.6%] vs 14.5% [95% CI, 13.7%-15.4%]) and receive income through social security or disability (14.0% [95% CI, 12.9%-15.2%] vs 5.3% [95% CI, 4.9%-5.7%]). The most common childhood conditions were ADHD (8.6%; 95% CI, 8.2%-9.0%), asthma (7.0%; 95% CI, 6.7%-7.3%), and learning disability (6.3%; 95% CI, 5.9%-6.6%).

**Table 1.** Sample Characteristics

Characteristics	Children, % (95% CI)		
	Total sample	With chronic conditions^a^	With no chronic conditions^a^
Unweighted sample, No.	33 187	6942	26 176
Weighted sample, No.	63 163 342	12 915 671	50 120 942
Sex			
Female	51.1 (50.5-51.7)	61.1 (59.7-62.5)	48.5 (47.8-49.2)
Male	48.9 (48.3-49.6)	38.9 (37.6-40.3)	51.5 (50.8-52.2)
Race or ethnicity			
Non-Hispanic American Indian or Alaskan Native	1.6 (1.2-2.3)	1.9 (1.3-2.8)	1.5 (1.1-2.2)
Non-Hispanic Asian	4.5 (4.1-4.9)	2.1 (1.7-2.5)	5.1 (4.7-5.6)
Non-Hispanic Black or African American	12.4 (11.5-13.4)	15.6 (14.2-17.1)	11.6 (10.7-12.6)
Hispanic, any race	25.7 (24.0-27.5)	22.9 (21.0-25.0)	26.4 (24.7-28.3)
Non-Hispanic White	51.6 (49.9-53.3)	53.1 (51.1-55.1)	51.2 (49.5-52.9)
Other single and multiple races	4.2 (3.8-4.5)	4.4 (3.8-5.0)	4.1 (3.7-4.5)
Age group, y			
2-4	17.7 (17.2-18.2)	9.2 (8.4-10.0)	19.9 (19.3-20.5)
5-9	31.0 (30.4-31.7)	28.4 (27.1-29.7)	31.7 (31.0-32.5)
10-14	31.9 (31.3-32.5)	38.1 (36.8-39.5)	30.3 (29.6-31.0)
15-17	19.4 (18.9-19.9)	24.3 (23.2-25.5)	18.1 (17.6-18.7)
Family income, % of FPL			
0-99	15.6 (14.8-16.5)	20.0 (18.6-21.6)	14.5 (13.7-15.4)
100-199	22.9 (22.1-23.6)	24.5 (23.2-25.9)	22.4 (21.6-23.3)
200-399	29.4 (28.7-30.2)	27.3 (26.0-28.6)	30.0 (29.2-30.8)
≥ 400	32.1 (31.0-33.2)	28.2 (26.7-29.7)	33.1 (31.9-34.3)
Highest educational level in family			
Less than high school	5.8 (5.4-6.4)	5.8 (5.0-6.7)	5.9 (5.4-6.4)
High school or graduate equivalent	17.4 (16.7-18.1)	18.8 (17.6-20.1)	17.0 (16.3-17.8)
More than high school	76.7 (75.8-77.6)	75.3 (73.9-76.7)	77.0 (76.0-78.0)
Children in family			
1	21.7 (21.3-22.2)	24.6 (23.6-25.6)	21.0 (20.6-21.5)
2	39.3 (38.6-40.0)	38.8 (37.4-40.2)	39.4 (38.6-40.2)
≥3	38.9 (38.1-39.8)	36.7 (35.1-38.3)	39.5 (38.6-40.5)
Employed adults in family			
0	6.6 (6.2-7.0)	10.9 (10.0-12.0)	5.5 (5.1-5.9)
1	37.4 (36.7-38.1)	38.6 (37.2-40.0)	37.1 (36.3-37.9)
2	47.6 (46.7-48.4)	42.7 (41.2-44.3)	48.8 (47.9-49.7)
≥3	8.5 (8.1-8.9)	7.7 (7.0-8.6)	8.7 (8.2-9.2)
Receipt of SSI or SSDI			
Yes	7.1 (6.6-7.5)	14.0 (12.9-15.2)	5.3 (4.9-5.7)
No	92.9 (92.5-93.4)	86.0 (84.8-87.1)	94.8 (94.4-95.1)
Insurance coverage type			
Private only	55.5 (54.2-56.7)	48.7 (47.0-50.4)	57.3 (56.0-58.6)
Public only	36.8 (35.6-38.0)	45.2 (43.5-46.8)	34.6 (33.3-36.0)
Other coverage	3.2 (2.7-3.7)	3.3 (2.7-4.1)	3.1 (2.7-3.6)
Uninsured	4.6 (4.2-5.0)	2.9 (2.4-3.4)	5.0 (4.6-5.4)

Abbreviations: FPL, federal poverty limit; SSDI, Social Security Disability Insurance; SSI, Supplemental Security Income.

^a^ Chronic conditions included asthma, attention-deficit**/**hyperactivity disorder, autism spectrum disorder, developmental delay, intellectual disability, learning disability, and prediabetes or diabetes.

### Prevalence of Food Insecurity in Children With Any vs No Chronic Condition

Across the study period, the weighted prevalence of food insecurity among all children in the sample was 10.2% (95% CI, 9.7%-10.7%). The prevalence of food insecurity among children with and without any of the 7 chronic conditions was 14.8% (95% CI, 13.7%-16.0%) and 9.0% (95% CI, 8.5%-9.5%), respectively. After adjusting for family and child characteristics in the pooled cross-sectional analysis, the higher prevalence of food insecurity among children with any chronic condition attenuated but persisted (AME, 2.6 percentage points; 95% CI, 1.7 to 3.5 percentage points).

The magnitude of the prevalence of food insecurity among children with and without chronic conditions varied over time, as did the difference in these prevalences (Figure). In 2019, the weighted prevalence of food insecurity among children with and without any of the 7 chronic conditions was 16.3% (95% CI, 14.1%-18.8%) and 9.7% (95% CI, 8.7%-10.8%), respectively. In 2021, the corresponding quantities were 9.7% (95% CI, 8.1%-11.7%) and 6.6% (95% CI, 5.8%-7.6%), respectively, but these quantities increased to 15.9% (95% CI, 13.9%-18.2%) and 11.1% (95% CI, 10.1%-12.1%) by 2023.

**Figure.**
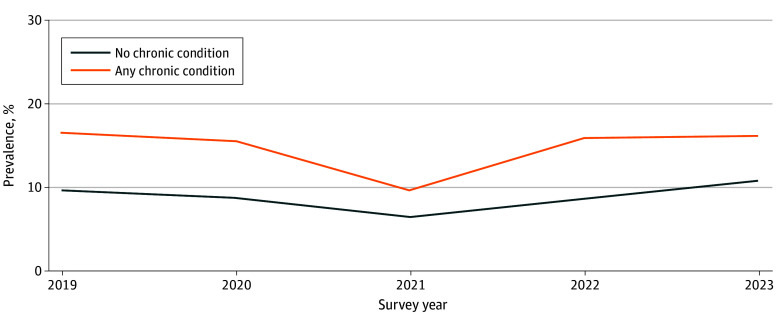
Annual Weighted Prevalence of Food Insecurity Among Children With and Without Chronic Conditions, NHIS 2019-2023

### Subgroup Analyses

Among children with each of the 7 chronic conditions, the prevalence of food insecurity was higher compared with those without any of the chronic conditions. Prevalence was highest in children with prediabetes or diabetes (21.8%; 95% CI, 17.3%-27.0%) and lowest in children with ADHD (14.4%; 95% CI, 12.8%-16.1%). In adjusted models, 6 of the 7 chronic conditions were associated with a higher probability of food insecurity (Table 2). The marginal effect was largest for prediabetes or diabetes (AME, 4.7 percentage points; 95% CI, 2.0 to 7.4 percentage points), followed by intellectual disability (AME, 3.6 percentage points; 95% CI, 1.0 to 6.2 percentage points). The AME was smallest for learning disability (1.8 percentage points; 95% CI, 0.5 to 3.1 percentage points). The association was positive but not significant for developmental delay (AME, 1.3 percentage points; 95% CI, −0.4 to 3.0 percentage points).

**Table 2.** Association of Childhood Conditions With Food Insecurity in the National Health Interview Survey, 2019-2023

Condition	Weighted frequency of food insecurity	Weighted prevalence of food insecurity, % (95% CI)	Average marginal effect (95% CI)
**Primary analysis**			
Children without a chronic condition	4 511 011	9.0 (8.5-9.5)	[Reference]
Children with any of the 7 chronic conditions	1 912 812	14.8 (13.7-16.0)	2.6 (1.7 to 3.5)
**Subgroup analysis: children with specific conditions vs none**			
Children without a chronic condition	4 511 011	9.0 (8.5-9.5)	[Reference]
Asthma	751 576	17.0 (15.2-19.1)	3.2 (1.9 to 4.6)
Attention-deficit/hyperactivity disorder	778 537	14.4 (12.8-16.1)	1.9 (0.5 to 3.2)
Autism spectrum disorder	327 199	16.3 (13.8-19.2)	2.2 (0.4 to 4.0)
Developmental delay	377 282	15.0 (12.8-17.6)	1.3 (−0.4 to 3.0)
Intellectual disability	167 044	20.1 (15.9-24.9)	3.6 (1.0 to 6.2)
Learning disability	692 493	17.5 (15.6-19.5)	1.8 (0.5 to 3.1)
Prediabetes or diabetes	152 071	21.8 (17.3-27.0)	4.7 (2.0 to 7.4)
**Subgroup analysis: children with 1, 2, or 3 chronic conditions vs none**			
Children without a chronic condition	4 511 011	9.0 (8.5-9.5)	[Reference]
Children with 1 chronic condition	1 137 405	13.2 (11.9-14.7)	2.2 (1.0 to 3.4)
Children with 2 conditions	437 216	16.9 (14.6-19.4)	3.9 (1.9 to 5.9)
Children with 3 or more conditions	338 190	19.5 (16.6-22.8)	3.0 (0.8 to 5.2)

The weighted prevalence of food insecurity among children with 1, 2, or 3 or more chronic conditions was 13.2% (95% CI, 11.9%-14.7%), 16.9% (95% CI, 14.8%-19.4%), and 19.5% (95% CI, 16.6%-22.8%), respectively. In adjusted comparisons, having 1, 2, or 3 or more chronic conditions was associated with a higher prevalence of food insecurity compared with having none (1 condition: AME, 2.2 percentage points; 95% CI, 1.0 to 3.4; 2 conditions: AME, 3.9 percentage points; 95% CI, 1.9 to 5.9 percentage points; 3 or more conditions: AME, 3.0 percentage points; 95% CI, 0.8 to 5.2 percentage points).

### Supplemental Analyses

When limiting the sample to children who could be linked to the sample adult in the household, the sample size decreased to 28 796 children, corresponding to a weighted sample of 54 721 340 children. When controlling for the presence of 1 or more chronic adult conditions in the sample adult, the association between having any chronic condition and food insecurity remained positive and significant (AME, 2.1 percentage points; 95% CI, 1.1 to 3.0 percentage points). In subgroup analyses, the association between individual chronic conditions and food insecurity remained significant for 3 of the 7 conditions (eTable 4 in Supplement 1).

When limiting the sample to eligible children ages 5 to 17 years, the sample decreased to 27 413 children, corresponding to a weighted sample of 51 796 449 children. The weighted prevalence of daily anxiety and depression symptoms were 5.7% (95% CI, 5.4%-6.1%) and 1.5% (95% CI, 1.3%-1.7%), respectively. The weighted prevalence of food insecurity in children with anxiety symptoms and children with depression symptoms was 17.0% (95% CI, 14.7%-19.6%) and 21.5% (95% CI, 17.1%-26.7%), respectively. Having any of the 9 chronic conditions (the 7 in the main analysis, and children with anxiety or depression symptoms) was associated with a higher prevalence of food insecurity (AME, 2.7 percentage points; 95% CI, 1.7 to 3.8 percentage points). Children with anxiety symptoms had a higher adjusted prevalence of food insecurity compared with those with none of the 9 chronic conditions (AME, 3.8 percentage points; 95% CI, 2.1 to 5.4 percentage points). The same was true for children with depression (AME, 4.2 percentage points; 95% CI, 1.5 to 6.8 percentage points) (eTable 5 in Supplement 1).

## Discussion

This analysis of a nationally representative survey of child health revealed that food insecurity is more common in children with any of 7 chronic conditions compared with children without these chronic conditions. This difference attenuated but persisted even when accounting for potential confounders of the association between chronic conditions and food insecurity, such as income.

To our knowledge, this is the first nationally representative analysis in children examining the association between having a broad range of common chronic conditions and household food insecurity. Our findings add to prior research that has examined the association between food insecurity and chronic conditions in children.^3,16,17,18,19^ Our findings are consistent with prior studies in adults demonstrating a higher prevalence of food insecurity among those with chronic conditions.^10,20^ Our study is consistent with prior adult studies demonstrating that chronic medical conditions are associated with food insecurity even when controlling for potentially important confounders.^21,22,23^ Our study adds to this research by extending these findings to children using a nationally representative survey.

The higher unadjusted prevalence of food insecurity among children with chronic conditions has important implications for clinical practice. First, clinicians in settings that do not universally screen for food insecurity could prioritize this screening when caring for children with chronic conditions. Second, pediatric subspecialty clinics may wish to consider implementing food insecurity screening as these clinics are frequent sites of care for children with chronic conditions—including those with multiple chronic conditions, a group that had a particularly high rate of food insecurity in this analysis. In addition to increased screening for food insecurity, it is also important to develop processes for connecting children who screen positive to programs that can provide food, such as food banks. Developing such processes, as well as integrating screening for food insecurity into clinical workflows, will require investing resources. Research is needed to determine the most efficient method to achieve these goals.

Our findings also have policy implications. They suggest that efforts to enroll children in policy interventions to prevent or mitigate the adverse health effects of food insecurity are particularly important for those with chronic conditions. For example, several state Medicaid programs are piloting Section 1115 waivers to design programs to address health-related social needs, the most common of which is food insecurity.^8^ In light of our findings, Medicaid programs might devote resources to ensuring that eligibility for these programs is consistently assessed for children with chronic conditions during health care visits. Additionally, many health insurers and health systems are investing in Food is Medicine programs. Given our findings, targeting these Food is Medicine programs to families with children with chronic conditions may be particularly important to reduce the prevalence of food insecurity, especially for families who are not eligible for benefits, such as SNAP or WIC, or those who continue to be food insecure despite receiving these benefits.^9^ Despite the promise of these emerging interventions, program access is variable across states, which highlights the critical role of protecting and strengthening national policy efforts to mitigate food insecurity in children. Our findings indicate that the association between having a chronic condition and food insecurity in children is only partly explained by differences in family and child demographic characteristics. Some of this association may be explained by the presence of unmeasured confounding factors associated with both chronic conditions and food insecurity that could not be accounted for due to the cross-sectional design of our analysis. Examples of a possible unobserved confounder include neighborhood characteristics,^24,25,26^ the built environment,^27^ and receipt of other nutrition programs, such as the School Breakfast Program or the National School Lunch Program.^28^

It is also possible that chronic conditions in children are causally linked to food insecurity. To the degree that this causal link exists, it is likely bidirectional.^29^ On the one hand, chronic conditions could increase food insecurity. Parents of children with these conditions frequently miss work to attend to their child’s needs, decreasing their economic productivity.^30^ Additionally, these parents also face high direct costs for their child’s care.^31,32^ These factors may contribute to family resource strain and subsequent food insecurity.

On the other hand, food insecurity may lead to the development of chronic conditions. For instance, prior research suggests that food insecurity may worsen diet quality and increase chronic stress, which in turn could increase the risk of prediabetes, diabetes, or other cardiometabolic conditions.^33,34,35^ Prior studies also suggests that food insecurity may also worsen mental health,^36,37,38^ potentially explaining the positive association between having anxiety and depression symptoms and food insecurity in one of our supplemental analyses. Further research is needed to better elucidate the mechanism of the persistent association between chronic conditions and food insecurity demonstrated in this study,

The prevalence of food insecurity decreased during 2021 among all children, regardless of whether they had chronic conditions or not. This finding parallels the findings of prior population-based studies^1^ and is likely attributable to interventions implemented during the COVID-19 pandemic, such as stimulus checks, the expanded child tax credit, and increased eligibility and benefits for SNAP and WIC.^39,40,41^ Notably, the gap in the prevalence of food insecurity between children with and without chronic conditions narrowed during 2021, suggesting that these interventions may have been particularly beneficial to children with chronic conditions.

However, by 2023 the prevalence of food insecurity among children with and without chronic conditions had returned to levels similar to those during 2019. This finding suggests that the decrease in food insecurity during 2021 was only temporary, and that the subsequent return to prepandemic levels of food insecurity was partly due to the expiration of pandemic-related supportive policies described above. Moreover, both the level and the difference in the prevalence of food insecurity between children with and without chronic conditions was smaller in 2021 than in other years. Had this anomalous year been excluded, the level and difference in the prevalence of food insecurity that we report would have been larger.

### Limitations

Our study has several limitations. First, the cross-sectional nature of this study limits our ability to evaluate whether the association between chronic childhood conditions and food insecurity was causal or explore the direction of any causal link. Second, we did not evaluate the association between obesity and food insecurity, as the NHIS only included questions on obesity in 2020 and 2022, limiting sample sizes. Third, we were able to control for primary insurance type, but data limitations precluded us from also controlling for whether patients had secondary sources of insurance coverage. Finally, our definitions of childhood chronic conditions were limited by available survey items in NHIS.

## Conclusions

Children with chronic conditions are more likely to have food insecurity compared with those without chronic conditions. Future research should explore the directionality and mechanism of the association between chronic conditions and food insecurity, as well as the degree to which this association varies according to the intensity of food insecurity. Future studies should also investigate the prevalence of food insecurity among specific subgroups of children with chronic conditions, such as children with obesity or children with medical complexity.

## Notes

Supplement 1. **eTable 1.** NHIS Questions Used in Study **eTable 2.** Chronic Condition Prevalence in Sample Population **eTable 3.** Frequency of Missing Values for Variables Included in Analysis **eTable 4.** Supplemental Analysis Including Adult Chronic Conditions as a Covariate **eTable 5.** Supplemental Analysis Including Children With Depression and Anxiety Symptoms

Supplement 2.
Data Sharing Statement
