# Supplementary material for: Chronic Conditions and Food Insecurity in US Children
Source: JAMA Netw Open. 2025 Sep 26;8(9):e2533953. doi: 10.1001/jamanetworkopen.2025.33953 (PMC12475946; doi:10.1001/jamanetworkopen.2025.33953)
Supplement: Supplement 1. — eTable 1. NHIS Questions Used in Study eTable 2. Chronic Condition Prevalence in Sample Population eTable 3. Frequency of Missing Values for Variables Included in Analysis eTable 4. Supplemental Analysis Including Adult Chronic Conditions as a Covariate eTable 5. Supplemental Analysis Including Children With Depression and Anxiety Symptoms [file jamanetwopen-e2533953-s001.pdf]

Supplemental Online Content

Hill NE, Palakshappa D, Chua K. Chronic conditions and food insecurity in US children. *JAMA Netw Open.* 2025;8(9):e2533953. doi:10.1001/jamanetworkopen.2025.33953

eTable 1. NHIS Questions Used in Study

eTable 2. Chronic Condition Prevalence in Sample Population

eTable 3. Frequency of Missing Values for Variables Included in Analysis

eTable 4. Supplemental Analysis Including Adult Chronic Conditions as a Covariate

eTable 5. Supplemental Analysis Including Children With Depression and Anxiety Symptoms

This supplemental material has been provided by the authors to give readers additional information about their work.

© 2025 Hill NE et al. *JAMA Network Open*.

eTable 1. NHIS questions used in study

Variable	Question	Age range
Anxiety symptoms	How often does [child name] seem very anxious, nervous, or worried? Would you say: daily, weekly, monthly, a few times a year, or never?	5-17
Asthma	Does [child name] still have asthma?	0-17
ADHD	Does [child name] currently have Attention-Deficit/Hyperactivity Disorder or ADHD or Attention-Deficit Disorder or ADD?	2-17
Autism spectrum disorder	Does [child name] currently have Autism, Asperger's disorder, pervasive developmental disorder, or autism spectrum disorder?	2-17
Depression symptoms	How often does [child name] seem very sad or depressed? Would you say: daily, weekly, monthly, a few times a year, or never?	5-17
Developmental delay	Does [child name] still have this other developmental delay?	0-17
Intellectual disability	Does [child name] currently have an intellectual disability, previously known as mental retardation?	0-17
Learning disability	Does [child name] currently have a learning disability?	2-17
Prediabetes	Has a doctor or other health professional ever told you that [child name] had prediabetes or borderline diabetes?	0-17
Diabetes	Has a doctor or other health professional ever told you that [child name] had diabetes?	
1 Positive if (1) daily<br>2 Combined into one indicator variable		

eTable 2. Chronic condition prevalence in sample population

Variable	Weighted Prevalence of Condition, % (95% CI)
Anxiety symptoms[1]	5.7 (5.4, 6.1)
Asthma	7.0 (6.7, 7.3)
ADHD	8.6 (8.2, 9.0)
Autism spectrum disorder	3.2 (2.9, 3.4)
Depression symptoms[1]	1.5 (1.3, 1.7)
Developmental delay	4.0 (3.7, 4.3)
Intellectual disability	1.3 (1.2, 1.5)
Learning disability	6.3 (5.9, 6.6)
Prediabetes or Diabetes	1.1 (1.0, 1.3)

© 2025 Hill NE et al. *JAMA Network Open*.

[1] Prevalence estimates reflect children ages 5-17

eTable 3. Frequency of missing values for variables included in analysis

	Frequency (%)
Health conditions	
Ages 2-17 years:[1]	
Asthma	64 (0.19)
Attention-deficit hyperactivity disorder	123 (0.36)
Autism spectrum disorder	71 (0.21)
Developmental delay	53 (0.16)
Intellectual disability	52 (0.15)
Learning disability	96 (0.28)
Prediabetes or Diabetes	31 (0.09)
Ages 5-17 years:	
Anxiety symptoms	80 (0.28)
Depression symptoms	78 (0.28)
Covariates[1]	
Child age	0 (0.0)
Child sex	14 (0.04)
Child race and ethnicity	0 (0.0)

© 2025 Hill NE et al. *JAMA Network Open*.

Health insurance type	133 (0.39)
Highest family educational attainment	0 (0.0)
Family income relative to federal poverty level	0 (0.0)
Receipt of income from SSI OR SSDI	219 (0.65)
Number of children in family	21 (0.06)
Census Region	0 (0.0)
Rurality	0 (0.0)
Number of parents in family	21 (0.06)
Number of employed adults in family	292 (0.85)
[1] Children ages 0-2 were excluded from primary analysis given that not all chronic conditions were assessed in children under age 2.	

eTable 4. Supplemental analysis including adult chronic conditions as a covariate

Condition	Weighted Frequency of Food Insecurity	Weighted Prevalence of Food Insecurity, % (95% CI)	Average marginal effect (95% CI)
Primary analysis			
Children without a chronic condition	3,884,695	9.0 (8.4, 9.5)	Reference
Children with any of the 7 chronic conditions	1,682,293	14.9 (13.8, 16.1)	2.1 (1.1, 3.0)
Subgroup analysis: children with specific conditions vs none			
Children without a chronic condition	3,884,695	9.0 (8.4, 9.5)	Reference
Asthma	658,560	17.3 (15.2, 19.6)	2.7 (1.3, 4.2)
Attention-deficit hyperactivity disorder	689,872	14.4 (12.7, 16.3)	1.3 (-0.06, 2.7)
Autism spectrum disorder	277,860	15.5 (12.8, 18.5)	1.3 (-0.7, 3.2)
Developmental delay	321,904	14.4 (12.1, 17.1)	0.5 (-1.4, 2.3)
Intellectual disability	149,825	20.5 (16.0, 25.9)	3.9 (1.0, 6.8)
Learning disability	600,285	17.5 (15.5, 19.8)	1.3 (-0.1, 2.7)
Prediabetes or Diabetes	129,461	21.7 (16.8, 27.4)	4.4 (1.6, 7.3)

eTable 5. Supplemental analysis including children with depression and anxiety symptoms

Condition	Weighted Frequency of Food Insecurity	Weighted Prevalence of Food Insecurity, % (95% CI)	Average marginal effect (95% CI)
Primary analysis*			
Children without a chronic condition	3,375,938	8.7 (8.2, 9.3)	Reference
Children with any of the 9 chronic conditions	1,908,114	14.6 (13.5, 15.7)	2.7 (1.7, 3.8)
Subgroup analysis: children with specific conditions vs none*			
Children without a chronic condition	3,375,938	8.7 (8.2, 9.3)	Reference
Anxiety symptoms	505,408	17.0 (14.7, 19.6)	3.8 (2.1, 5.4)
Asthma	687,737	17.1 (15.1, 19.3)	3.0 (1.7, 4.4)
Attention-deficit hyperactivity disorder	756,650	14.3 (12.7, 16.1)	1.9 (0.6, 3.2)
Autism spectrum disorder	291,305	16.9 (14.2, 20.0)	2.9 (0.9, 4.9)
Depression symptoms	167,117	21.5 (17.1, 26.7)	4.2 (1.5, 6.8)
Developmental delay	286,655	15.1 (12.6, 17.9)	1.0 (-1.0, 2.9)
Intellectual disability	155,590	20.9 (16.5, 26.2)	4.2 (1.4, 6.9)
Learning disability	657,471	17.7 (15.7, 19.8)	2.0 (0.6, 3.3)
Prediabetes or Diabetes	151,376	22.4 (17.8, 27.8)	5.0 (2.3, 7.6)
* Only includes children ages 5-17 because anxiety and depression symptoms were not surveyed in children under 5 years.			

© 2025 Hill NE et al. *JAMA Network Open*.
