# Supplementary material for: Chronic Conditions and Food Insecurity in US Children
Source: JAMA Netw Open. 2025 Sep 26;8(9):e2533953. doi: 10.1001/jamanetworkopen.2025.33953 (PMC12475946; doi:10.1001/jamanetworkopen.2025.33953)
Supplement: Supplement 2. — Data Sharing Statement [file jamanetwopen-e2533953-s002.pdf]

Data Sharing Statement

Hill. Chronic Conditions and Food Insecurity in US Children. *JAMA Netw Open*. Published September 26, 2025. doi:10.1001/jamanetworkopen.2025.33953

Data
Data available: No

Additional Information
Explanation for why data not available: Data used for study is publicly available through CDC National Center for Health Statistics, National Health Interview Survey
